# The mental health of nurses in acute teaching hospital settings: a cross-sectional survey

**DOI:** 10.1186/s12912-015-0068-8

**Published:** 2015-03-27

**Authors:** Lin Perry, Scott Lamont, Scott Brunero, Robyn Gallagher, Christine Duffield

**Affiliations:** 1 Faculty of Health, University of Technology Sydney, Ultimo, NSW 2007 Australia; 2 Mental Health Liaison, Prince of Wales Hospital and Community Health Services, Randwick, NSW 2031 Australia; 3 Charles Perkins Centre and Sydney Nursing School, University of Sydney, Sydney, NSW 2006 Australia; 4 Centre for Health Services Management, Faculty of Health, University of Technology Sydney, Ultimo, NSW 2007 Australia; 5 Edith Cowan University, Perth, WA Australia

**Keywords:** Nursing workforce, Nurses, Mental health, Vitality, Common mental disorder, Depression, Anxiety, Absenteeism, Presenteeism

## Abstract

**Background:**

Nursing is an emotionally demanding profession and deficiencies in nurses’ mental wellbeing, characterised by low vitality and common mental disorders, have been linked to low productivity, absenteeism and presenteeism. Part of a larger study of nurses’ health, the aim of this paper was to describe the mental health status and related characteristics of nurses working in two acute metropolitan teaching hospitals.

**Methods:**

A cross sectional survey design was used.

The Registered and Enrolled Nurse workforce, employed on any form of contract, at two teaching hospitals in Sydney Australia were invited to participate. The survey tool was compiled of validated tools and questions. Family and medical history and health risk-related characteristics, current psycho-active medications, smoking status, alcohol intake, eating disorders, self-perceived general health, mental health and vitality, demographic, social and occupational details were collected.

**Results:**

A total of 1215 surveys were distributed with a usable response rate of 382 (31.4%). Altogether 53 nurses (14%) reported a history of mental health disorders, of which n = 49 (13%) listed diagnoses of anxiety and/or depression; 22 (6%) were currently taking psychoactive medication. Symptoms that could potentially indicate a mental health issue were more common, with 248 (65.1%) reporting they had experienced symptoms sometimes or often in the last 12 month.

Nurses had better mental health if they had better general health, lived with a spouse/ partner rather than alone, had fewer symptoms, sleep problems or disordered eating behaviours, were not an informal carer and did not work nights. Nurses had greater vitality if they were male, had better general health, fewer sleep problems or symptoms generally and lived with a spouse/ partner rather than alone; less vitality if they were an informal carer or had disordered eating.

**Conclusion:**

Nurses and their managers should strive to create workplaces where working practices promote nurses’ health and wellbeing, or at least are configured to minimise deleterious effects; where both nurses and their managers are aware of the potential for negative effects on the mental health of the workforce; where cultures are such that this can be discussed openly without fear of stigma or denigration.

## Background

Internationally, increasing demands for healthcare combine with workforce shortages to make it imperative to identify strategies to promote staff retention and productivity in the health workforce. Nurses comprise the largest single professional group in the health workforce [1] but the average age of nurses in developed countries is increasing. Nursing is an emotionally and physically demanding occupation and with research indicating that nursing work entails high risks of experiencing stress, anxiety and depression [2,3], ensuring the health of nurses is important to safeguard workforce supply.

Anxiety and depressive disorders are known as common mental disorders (CMD) [4]; CMD, substance abuse, workplace aggression, stress and burnout (common precursors of CMD) have all been reported in the nursing occupational health literature [5-7]. Burnout and low vitality have repeatedly been reported amongst nurses [7-9]. In this context vitality is defined as a sense of general wellbeing, optimism and flourishing, and can be considered to represent the opposite end of a spectrum to stress and burnout [10]. Low vitality and CMDs may result from a variety of workplace, organisational and individual factors [11,12]. These include working environments where nurses lack autonomy and discretion, and where access to support and learning is limited [13]; where high levels of emotional exhaustion and burnout occur [14]; where nurses experience frequent workplace stress [15,16], high workloads and low reward [17], and where rotational shift patterns result in poor sleep patterns [18]. Mental health repercussions may occur where nurses are subjected to high public and professional expectations [19], workplace violence [5,20] and role conflict [21]; in environments where there is high patient mortality, traumatic events or situations [22] and conflict with physicians [23].

Beyond the importance to the individuals involved, in the nursing workforce low vitality and high CMDs have implications for patient care. They have been strongly associated with nursing productivity [24], absenteeism and presenteeism (attending work while sick) [25]; with poor work performance, reduced productivity, workplace errors, decreased quality of patient care and low levels of patient satisfaction [26]. It is critical that employers understand issues relating to mental health, wellbeing and vitality in the nursing workforce to enhance performance and prevent compromise to care delivery. A crucial step in addressing low vitality and CMDs is early identification of unhealthy behaviours, health symptoms and complaints. This will enable interventions to be developed to promote mental wellbeing, flourishing and vitality in the nursing workforce [27]. Encouragement of healthy habits in the nursing workforce may facilitate workplace cultures grounded in positive energy and vitality [28]. This study entails first steps in developing understanding of mental health and wellbeing in the nursing workforce.

## Methods

### Aim

Part of a larger study of nurses’ health, the aim of this component was to determine the mental health status and related characteristics of nurses working in two acute metropolitan tertiary referral hospitals in Sydney, Australia.

### Study design

This study used a cross sectional survey design. Guided by the processes of Intervention Mapping [29], material from a series of literature reviews of the current state of knowledge of health promotion, lifestyle and behavioural interventions for women and nurses was used to identify survey elements, variables and items to map behavioural health risk factors of working nurses. This paper reports only mental health –related components.

### Sample

All Registered Nurses (RNs) and Enrolled Nurses (ENs; second level nurses who work in an associate role alongside Registered Nurses) employed on any form of contract at the two study sites were invited to participate. The workforce totalled 1,270 and 232 nurses, respectively, but some nurses were on leave and unavailable during the study period.

### Survey instrument

The survey tool was compiled of validated tools and questions. Family and medical history and health risk-related characteristics were sought using the formats employed by the Australian Longitudinal Study of Women’s Health (ALSWH; see http://www.alswh.org.au/) and the Health in Men Study (HIMS, see http://www.wacha.org.au/hims.html), including current psycho-active medications, tobacco and alcohol use. Diagnoses and self-reported symptoms potentially reflective of physical and mental ill health were obtained. These survey formats were chosen as ALSWH and HIMS are established, long-running studies including health characteristics mainly derived from previously validated instruments with demonstrated rigor. Demographic, social and occupational details were also collected.

Self-perceived general health, mental health and vitality were examined using the relevant subscales of the Medical Outcomes Survey Short Form 36 (MOS SF-36) [30]. The MOS SF 36 has undergone extensive validation and reliability testing (see http://www.sf-36.org/tools/sf36.shtml#MODEL) and has been validated and used in the Australian Household, Income and Labour Dynamics in Australia Survey [31], providing Australian reference values.

A five-question screening tool for eating disorders was included (the SCOFF tool), which has demonstrated good concurrent and discriminant validity and reliability with clinical, community and student populations at a cut-off of two or more positive responses [32-35].

Sleeping problems were determined using the abbreviated form of the Insomnia Severity Index [36] used by ALSWH. Questions from the ALSWH and Tucker et al [37] were used to determine a history of workplace injury and abuse.

### Data collection

Letters of invitation, Information Statements and copies of the survey were delivered and returned through the internal hospital post; reminders were delivered by email. Return of a completed survey was understood to convey consent to participate in this study. Data were collected over four months in 2011-2012 at the two sites.

### Data analyses

Data were entered and analysed using the Statistical Package for the Social Sciences (SPSS) for Windows Version 21. Descriptive analyses used means and standard deviations, frequency and percentage according to the level of the variable. The independent predictors of mental health and vitality (both composite scores of the relevant SF-36 domains) were determined using linear regression analyses. For both models forced entry was used of potentially explanatory variables suggested by relevant related literature: age, sex, social situation (living alone or with partner/ spouse), years of nursing experience, role autonomy (defined by the researchers as holding occupational positions allowing greater autonomy in work activities (educators, advanced practice nurses, managers) versus lesser autonomy (RNs and ENs)), fulltime employment, any history of mental health disorder, any prescription of psycho-active medications, working rotating rosters that included nights, primary language other than English (in recognition of the greater stresses accrued by migration [38,39], perceived general health, total number of symptoms potentially reflective of mental health, eating disorder risk score within ‘at risk’ category, any workplace injury, any workplace abuse, sleep issues score, moderate to high alcohol risk (weekly intake more than 14 drinks for women, 21 drinks for men, and/ or ‘binge’ drinking patterns entailing more than 5 drinks on one occasion at least once per month) and daily smoking [37,40-47]. The level of significance was set at 0.05.

### Ethical considerations

All participants were supplied with written information about the project and had the opportunity to ask questions of a local research team member. Surveys were completed anonymously; no identifying data were collected. As all participants had a current nursing qualification it was possible that in completing the survey, a participant might recognise that particular responses indicated a degree of personal risk to health. It was assumed that any such participant would use their professional knowledge to take appropriate steps to seek clarification of survey findings and would, in the first instance, consult their General Practitioner. As survey respondents were not identifiable, the research team were not able to respond to any such finding. Ethical approval was granted from the South Eastern Sydney Local Health District Human Research Ethics Committee, HREC reference 11/148 (LNR/11/POWH/242).

## Results

A total of 1,215 surveys were distributed with a usable response rate of 381 (31.4%), albeit with a few missing data points. Respondents had a mean age of 39.9 years with 45.3% aged 40 years and older; 24.4% aged 50 years and older. Approximately one quarter reported living alone. The sample were mostly female (89.5%), born outside of Australia (54.3%) (Table 1) and spoke English as their primary language (75.3%). With a mean of 14.7 years of experience in nursing, 40% had more than 16 years; 78.7% had at least a Bachelor degree and 36.7% had a postgraduate qualification. Almost half (47.8%) worked rotating shifts which included night shifts. Respondents worked in a diverse range of specialities: emergency departments, operating theatres and intensive care units (22.0%), outpatient patient departments and community outreach (29.1%) and medical/surgical wards (46.2%).

**Table 1:** **Socio-demographic and work characteristics**

Characteristic (n = 381)	Mean	SD
Age (range 20-67) years	39.9	11.7
	**n**	**%**
Female	315	82.7
Lives alone	96	25.2
**Country of birth** (n = 372)		
Australia	170	45.7
United Kingdom or Ireland	70	18.2
Asia	65	17.5
Europe	30	8.1
Other	39	10.5
**Work classification**		
*RN	216	56.7
*CNS/CNC/NP	84	22.1
*NM/NUM/Manager	41	10.7
*EN	18	4.7
*CNE/Educator	18	4.7
Other	4	1.1
**Work contract**		
Full time	303	79.6
Part-time (<1 FTE)	65	17.2
Casual/agency	12	3.2
**Highest qualification**		
Bachelor degree	160	42.0
Post-graduate certificate/diploma	84	22.0
Masters degree/ doctorate	56	14.7
Certificate only	41	10.8
Diploma only	36	9.5

*Registered Nurse/Clinical Nurse Specialist/Clinical Nurse Consultant/Nurse Practitioner/Nurse Manager/Nursing Unit Manager/Enrolled Nurse/Clinical Nurse Educator.

### Mental Health

**Figure 1:**
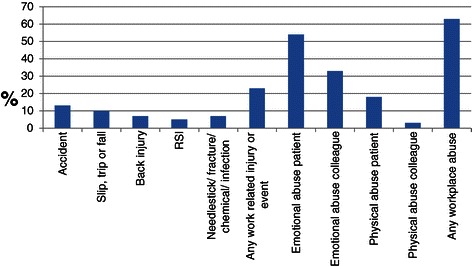
**Adverse work experiences in previous 12 months.** RSI: Repetitive strain injury.

Altogether 53 nurses (13.9%) reported a history of diagnosed mental health disorders with n = 49 (12.9%) listing anxiety/depression; 22 (5.8%) were currently taking a psychoactive medication. One fifth (n = 80, 21%) reported disordered eating behaviours (SCOFF scores **≥** 2), with n = 32 (8.4%) scoring **≥** 3 [35]. None of those whose scores indicated potential eating disorder identified psychiatric diagnoses other than anxiety or depression; of the ten who reported other psychiatric diagnoses, seven were in receipt of psychoactive medication. However, other symptoms that could potentially indicate a mental health issue were common, with 248 (65.1%) reporting they had experienced at least one of the seven listed symptoms sometimes or often in the last 12 months (Table 2), most commonly headaches and severe tiredness. Of the potential maximum of seven symptoms, respondents reported a mean (SD) of 1.6 (1.6) symptoms. In total n70 (18.2%) had sought help for these symptoms. The majority (71.5%) also reported at least one current sleep problem, most commonly waking in the early hours (n = 212, 57.3%). Adverse work-related experiences, including aggression and bullying, were reported by 62.8%, with 35.2% reporting two or more types (Figure 1). However, a median score of 4 was reported for perceived general health, indicating that overall these nurses thought their health was very good.

**Table 2:** **Symptoms potentially related to mental health issues experienced sometimes or often in last 12 months; whether help sought**

	Sometimes		Often		Sought help	
	n	%	n	%	n	%
Headaches	101	26.5	38	10.0	29	7.6
Severe tiredness	100	26.2	39	10.2	22	5.8
Indigestion/heartburn	50	13.1	15	3.9	22	5.8
Anxiety	45	11.8	12	3.1	22	5.8
Night sweats	27	7.1	19	5.0	13	3.4
Depression	29	7.6	12	3.1	24	6.3
Palpitations	31	8.1	4	1.0	2	0.5
**Sleep problems (current)**						
Waking in the early hours	212	55.6				
Sleeping badly at night	128	33.6				
Taking a long time to get to sleep	116	30.4				
Worrying keeping you awake at night	102	26.8				
Lying awake most of the night	70	18.4				
**Sleep problems score ≥ 1 (0-5)**	**268**	**70.3**				
**Eating disorders score ≥ 2 (0-5)**	**80**	**20.9**

### Tobacco and alcohol use

A minority of the group smoked cigarettes (18%), of whom 6.8% smoked every day (Table 3). Almost all nurses drank alcohol (92.5%) with 3.5% drinking every day and 34.8% drinking at least 5 alcoholic drinks in a day at least once a month. More than one third (39.2%) identified alcohol drinking habits that conferred moderate to high health risk.

**Table 3:** **Cigarette and alcohol use and level of risk**

Behaviour	n	%
**Cigarette smoking**		
Smokes	66	18.0
Smokes daily	25	6.8
**Alcohol use**		
Drinks	333	92.5
Drinks daily	13	3.5
≥5 drinks/day > once/month	128	34.8
Low risk category	177	47.6
Moderate to high risk category	146	39.2

### Predictors of mental health and vitality

Overall at 70.2 (SD 14.1), nurses’ mean mental health scores were lower than the Australian SF 36 normative reference score of 73.9 (17.4) for this domain; at 57.6 (16.3) mean scores on the vitality domain were also below the Australian SF 36 normative reference value of 60.9 (SD 19.9) [31]. Independent predictors of mental health and vitality were determined using linear regression. The model explained 34% of the variance in mental health with seven independent predictors (Table 4). Nurses had better mental health if they had better general health (β = 4.14); if they lived with a spouse or partner rather than alone (β = 7.35) or were not an informal carer (β = -5.59); if they had fewer symptoms (β = -2.06), sleep problems (β = -1.36) or disordered eating behaviours (β = -2.00); if they did not work nights (β = -3.04).

**Table 4:** **Predictors of mental health (SF36 Mental composite scale)**

Characteristics	Beta	95% confidence interval	P value
General health	3.17	2.68 – 6.15	<0.001
Live alone versus with partner	7.35	9.6 – 5.4	<0.001
Total sum of symptoms	-2.06	-4.0 – -0.3	0.001
Sleep problems, total	-1.36	-2.91 – -.0.86	0.006
Disordered eating	-2.00	-3.29 – -0.48	0.006
Informal carer role	-5.59	-7.24 – -3.1	0.025
Night shifts	-3.04	-2.53 – -5.54	0.04

Model statistics r^2^ = .340, f = 8.784, p <0.001.

The second model explained 24% of the variation in vitality with seven independent predictors (Table 5). Nurses had more vitality if they were male (β = -8.95), if they lived with a spouse or partner rather than alone (β = 4.68) or were not an informal carer (β = -7.89); if they had better general health (β = 4.14), fewer sleep problems (β = -1.67), disordered eating behaviours (β = -1.76) or symptoms (β = -1.91).

**Table 5:** **Predictors of vitality (SF36)**

Characteristics	Beta	95% confidence interval	P value
Sex	-8.95	-10.71 – -7.21	<0.001
General health	4.14	2.9 – 6.83	<0.001
Sleep problems, total	-1.67	-3.41 – -0.32	0.003
Total sum of symptoms	-1.91	-3.27 – -0.62	0.005
Informal carer role	-7.89	-9.27 – -4.62	0.006
Disordered eating score	-1.76	-3.04 – 0.103	0.034
Live alone v with partner	4.68	2.22 – 6.97	0.017

Model statistics r^2^ = .342, f = 8.915, p <0.001.

## Discussion

While nurses reported good general health overall a number of health issues were present, with vitality and mental health below Australian population values and evidence of poor health practice including misuse of alcohol, disordered eating behaviours and poor sleep. Better mental health and greater vitality were both reported in the presence of better self-reported general health, reinforcing the importance of good general health.

Low vitality and CMDs are crucial to consider, both for their impact on nurses themselves and on workforce participation. This study revealed significant clustering with night duty, poor sleep, disordered eating with higher levels of general symptoms and with living alone. Nurses’ absenteeism and presenteeism may result from low vitality and CMDs, which in this study were also linked to poor health behaviours. Absenteeism is commonly the primary concern in relation to organisational and workforce issues because it is easier to identify and measure. However presenteeism may be of even greater concern, due to the likelihood of its greater prevalence and implications for productivity, personal health and patient safety when nursing staff with CMDs remain at work [25]. A systematic review of sub-optimal work functions related to the presence of CMDs in nurses and allied health professionals identified increased incidence of general clinical errors, medication errors, near misses and decreased patient safety and patient satisfaction [4]. Low vitality may result in low productivity, possibly increasing the workload of colleagues or resulting in missed care.

Presenteeism amongst health professionals may account for 1.5 times more working time lost than absenteeism [48]. General population statistics in the US indicated 2.4 days lost from presenteeism reduced work performance, for each day related to absenteeism. In Australia, presenteeism amongst the general population has been estimated at almost four times that of absenteeism, at a cost of $25.7 billion for the years 2005/6 and $34.1 billion in 2009/2010 [49]. This reinforces the concern about the levels of CMDs and reduced vitality demonstrated in this study.

Greater symptom burden was linked with both reduced mental health and lower vitality (Tables 4 and 5), flagging the importance of considering nurses’ general health status and factors potentially influencing this. Symptoms with particularly high prevalence included sleep disturbance, headaches and severe tiredness. Shift work is well known to contribute adversely, across industries, to physical and mental health, job performance and psychological wellbeing; shift work and associated effects are particularly prevalent in healthcare workers [50]. For the nurse this translates to insufficient recovery time, poor sleep quantity and quality [51] and reduced physical and psychological wellbeing [52].

Whilst not shown as predictive of poorer mental health or vitality, the levels of alcohol use amongst these nurses was high. Rates of alcohol use that cause risk to health were seen in 39.2%, which is comparable to the short term/single occasion risk rate in the Australian general population [53]. This apparent equivalence is a particular concern in a workforce seen as the health educators and role models for the community [54], whose high health literacy should confer greater awareness of the deleterious effects of alcohol on their health and work performance than the general population.

However, dedicated alcohol reduction programmes for nurses are difficult to find. A synthesis of papers on substance abuse in the nursing profession concluded that poor or ineffective policies that mandate punitive action against nurses may endanger public health by making it difficult for impaired nurses to ask for help [55]. The authors argued for an early intervention model in helping nurses recover from addictive disorders, provided in a non-punitive, confidential atmosphere of support where recognition of nurses’ need for treatment is seen as their first step to rehabilitation.

The high levels of aggression-related events reported is unsurprising as this is recognised internationally as an increasing public health concern [56]. Workplace aggression and violence are known to lead to psychological trauma, post-traumatic stress, impaired performance and burnout [57,58]. Of concern is that participants in this study indicated a significant level of emotional abuse from colleagues. This may have been under-reported as this sort of abuse will primarily take the form of non-physical violence from managers or supervisors. It is also less likely to be reported as healthcare workers have a tendency to report only the most serious incidents [59]. Most literature has focused on patient-perpetrated incidents, and less on colleague related instances [60,61].

### Implications for nurses’ wellbeing and workforce health and safety

These study findings indicate some significant health vulnerability in the nursing workforce. Traditional approaches to managing nurses’ health issues have been largely reactive; that is, the provision of employee assistance programmes offering ‘after the fact’ follow up and counselling services [62]. The implications of this study are that the healthcare industry needs to be more proactive through providing wellness programs and employee screening and promoting awareness campaigns. Developing an increased level of mental health literacy in managers may provide a first level of detection for low vitality and CMDs in the workplace.

Several studies have explored mediating effects on nurses’ mental health. Mental health status as a component of job strain can be predictive of propensity to leave, with nurses’ mental health being favourably influenced by coping behaviours [63]. A number of strategies are available. The compatibility of a person’s characteristics with those of their work (‘person-environment fit’), for example, can moderate perceived stress, work-family conflict and mental health [64]; the negative relationship between work overload and mental health may be improved by intervention to reduce incivility and bullying behaviour in the workplace [65]; clinical supervision has been suggested to mediate nurse distress, stress and mental health [66] and reductions in psychological stress [67].

Efforts to address issues related to the presence of low vitality and CMD workforce issues face several barriers. These include perceived difficulty in replacement of staff and the perception amongst some that only they can do the work required [48]. Staff have a sense of loyalty to peers and lack of knowledge regarding their rights and related work scheduling options [68]. There are also associated stigma and the perception that experiencing CMDs is at odds with professional expectations and integrity. Further, emerging CMDs can be difficult to detect particularly in pre-clinical diagnostic stages, but efforts have been made to describe typical behaviours seen in people with emerging CMDs and substance abuse disorders in the work setting [26,55].

### Limitations

Study limitations include the response rate; whilst 31.4% was acceptable for a general survey, results should be interpreted with caution. It is possible that those positively inclined towards health issues participated in the study and were attitudinally and/or behaviourally different to those who chose not to participate. This was an inner metropolitan workforce, and their mean age (younger than the Australian nursing population age of 44.5 years), suggests this may not be a typical national sample: a larger representative study is clearly warranted. As with any self-report measures, respondents may have returned surveys with responses they thought were appropriate as opposed to a true reflection of their health status, and such ‘socially desirable responses’ may create false or obscure relationships in data. Social desirability responses may be more likely to occur with sensitive questioning such as the current study, where questions reflect aspects of wellbeing and health which nurses may not wish to confront [69]. However, this survey was anonymous, as a means to promote honest response. Finally, no information was sought about respondents’ pre-employment history or circumstances outside of the workplace. Findings are presented as description of the workforce rather than causally attributed to this employment.

## Conclusions

Nurses are a vital workforce for the health of the population. This study demonstrates that while nurses consider themselves to be generally healthy, there are several areas where, as a workforce, they could benefit from support, including management of fatigue through positive strategies such as self-rostering and sleep support programs. As the average age of a nurse participating in the workforce continues to increase it is likely that, without intervention, the health issues raised here will persist, hampering workforce participation and exacerbating projected shortages. Importantly, for patients, there may be serious consequences for their safety and for those nurses at work in less than ideal health. This aspect warrants further investigation. The costs of replacing a nurse who leaves because of ill health are considerable, as are the costs of lost productivity due to presenteeism or absenteeism. While nurses must take responsibility for their own health, the nature of nursing work is such that there is also an onus on employers to ensure they provide sufficient assistance and resources for staff.

The implications of study findings are that nurses and their managers should strive together to create workplaces where working practices promote nurses’ health and wellbeing, or at least are configured to minimise deleterious effects; where both nurses and their managers are aware of the potential for negative effects on the mental health of the workforce, and where cultures are such that this can be discussed openly without fear of stigma or denigration. Finally, both nurses and their managers need access to resources for timely, appropriate and proactive support to prevent unnecessary mental ill health and harms for the workforce and to preserve quality patient care.

## Abbreviations

ALSWH: Australian Longitudinal Study of Women’s Health
CMD: Common mental disorders
EN: Enrolled Nurses
HIMS: Health in Men Study
MOS SF-36: Medical Outcomes Survey Short Form 36
RN: Registered Nurses
SD: Standard deviation
SPSS: Statistical Package for the Social Sciences
